# Exploring the Association of Biochemical Characterization and Genetic Determinants of TNF-α, CXCR2, and CCR5 Delta 32 Mutation with Predisposition to Polycystic Ovary Syndrome

**DOI:** 10.3390/life14080949

**Published:** 2024-07-28

**Authors:** Kholoud S. Almasoudi, Eram Hussain, Reema Almotairi, Tanzeela Bhat, Nabil Mtiraoui, Intissar Ezzidi, Rashid Mir

**Affiliations:** 1 Department of Medical Laboratory Technology, Faculty of Applied Medical Sciences, Prince Fahad Bin Sultan Chair for Biomedical Research, University of Tabuk, Tabuk 71491, Saudi Arabia; kalmasoudi@ut.edu.sa (K.S.A.); e.husain@ut.edu.sa (E.H.); ralmotairi@ut.edu.sa (R.A.); tanzeelabhat01@gmail.com (T.B.); 2 Laboratory of Human Genome and Multifactorial Diseases, Faculty of Pharmacy, University of Monastir, Monastir 5000, Tunisia; mtiraouinabil@yahoo.fr (N.M.); iezzidi@ut.edu.sa (I.E.)

**Keywords:** polycystic ovary syndrome (PCOS), gene polymorphism, inflammatory markers, TNF-α, CCR5, CXCR2, amplification-refractory mutation system PCR (ARMS-PCR), IR-insulin resistance

## Abstract

PCOS is a heterogeneous, multifactorial endocrine disorder with a complex pathophysiology. It is a globally rising infertility disorder that affects a large percentage of women of reproductive age, with a relatively high prevalence of 8–13%. Genome-wide association studies have revealed associations of genetic variations with many diseases, including PCOS. The cellular activity of IL8 is mediated by the receptor CXCR2, and transcription of IL8 is controlled by TNF-α. Therefore, this study aimed to investigate the association of TNF-α, CCR5-delta32, and CXCR2 gene variations with PCOS. Methodology: In this case control study, we used amplification-refractory mutation system (ARMS)-PCR to detect and determine the presence of the polymorphic variants TNF-α, CCR5-delta32, and CXCR2 in the study subjects. These gene polymorphs may serve as critical candidate gene variants in PCOS pathogenesis and therapeutics. Results: The case–control study’s findings revealed that the majority of the biochemical and endocrine serum biomarkers examined in the investigation—including lipids (LDL, HDL, and cholesterol), T2DM markers (fasting glucose, free insulin, and HOMA-IR), and hormones (FSH, LH, testosterone, and progesterone)—exhibited statistically significant changes in PCOS patients. The distributions of TNF-α (rs1800629), CCR5-delta32, and CXCR2 (rs2230054) genotypes analyzed within PCOS patients and healthy controls in the considered population were significant (*p* < 0.05). The heterozygosity of CXCR2-CA, TNF-α GA, and CCR5(WT+Δ32*) genotypes was significantly associated with PCOS susceptibility, with high OR and *p* < 0.05 in the codominant model. Similarly, the A allele of the TNF-α and CXCR2 genes, along with the CCR5Δ32*(mutant) allele, was significantly associated with PCOS susceptibility, with high OR and *p* < 0.05. Likewise, the CXCR2 (CA+AA) vs CC genotype was associated with increased susceptibility to PCOS, with OR 2.25, *p* < 0.032. Conclusions: Our study concludes that TNF-α rs1800629G>A, CXCR2-rs2230054C>T, and CCR5-Delta32 rs333 are potential loci for developing PCOS in the Tabuk population. These findings might eventually be useful in identifying and classifying those who are at risk for PCOS. To validate these results, it is advised that further longitudinal studies be conducted in diverse ethnic populations and with larger sample sizes.

## 1. Introduction

Polycystic ovary syndrome (PCOS) is a common endocrine ailment in women that is characterized by hyperandrogenism, ovulatory disorder (such as menstrual disorder), and polycystic ovarian morphology (PCOM; a range of preantral follicles within the ovaries) [1]. The prevalence of PCOS in premenopausal women ranges from ~6% to ~20%, probably making this syndrome the most common endocrine and metabolic ailment in women of reproductive age [2,3]. Risk factors for PCOS are well established and include genetic elements, insulin resistance (IR), hyperinsulinemia, hyperandrogenism, ovarian follicular maturation arrest, gonadotropic derangements, obesity, etc. Indeed, a significant proportion of women with PCOS show metabolic abnormalities, including insulin resistance (IR), dyslipidemia, and obesity. Etiologically, PCOS arises from the complex interaction of gene variants with environmental factors [4]. PCOS is widely recognized as a proinflammatory condition in which ovarian and metabolic dysfunction have been reported to occur due to chronic inflammation of a low-grade nature. Using a thorough serum profile, this study describes the biochemical and endocrine parameters like BMI, IR, fasting insulin, homeostatic model assessment (HOMA), and lipid markers in serum in PCOS patients relative to healthy controls. Further, we investigated the effect of genotypic and allelic variants of CXCR2-rs2230054-C>T, TNF-α G>A (rs1800629) genes, and CCR5-Delta32 rs333 mutation on the susceptibility to PCOS. 

### 1.1. Tumor Necrosis Factor-Alpha (TNF-α) 

TNF-α, as a pro-inflammatory marker, is present in the follicular fluid of the ovary and oocytes with a dual role [5] and is required for follicular development, ovulation, and oocyte maturation [6,7]. Several reports highlight the involvement of various types of SNPs of TNF-α in PCOS, such as −1031 T>C (rs1799964) and rs1800629 [8]. One of the SNPs (rs1799964) has been significantly associated with PCOS, probably due to the altered promoter activity responsible for varying levels of TNF-α in the plasma of healthy individuals; however, due to a relatively small sample size, the rs1800629 variant of TNF-α has yielded conflicting results [9]. The rs1800629 SNP has been reported to have a significant effect on TNF-α transcriptional activity and regulate the expression level of TNF-α. Increased TNF-α can promote insulin resistance and cause hyperandrogenism. TNF-α represents one of the potent cytokines mediating pro-inflammatory and immunoregulatory roles with related linkage between HLA class I and class II genes. The influence of the −308 G>A polymorphism on the promoter activity of the TNF-α gene, along with the mapping of the gene within the major histocompatibility complex (MHC), may influence immunologic homeostasis and contribute to the pathogenesis of PCOS. The TNF-α 308 A allele is more involved in autoimmune diseases with higher transcriptional activity in comparison with the TNF-α-308G allele [10]. Several reports highlight the involvement of *TNF-*α (rs1800629) in the pathogenesis of POCS [11].

Effects of TNF-α on PCOS in animal models: The main modulator of TNF action in the mouse ovary is TNFR2. The increased TNFR2 concentrations associated with obesity and insulin resistance (IR) are influenced by a common polymorphism [12,13]. PCOS and hyperandrogenism in women have been linked to certain polymorphisms of TNF-α and its type 2 receptor, and individuals with PCOS have overexpressed TNFR2. TNF-α/TNFR2 and TLR4 are two main inflammatory pathways in the pathogenesis of PCOS. [14]. Therefore, it seems that inhibition of these inflammatory pathways can play an important role in treating the disease.

### 1.2. The CC Chemokine Receptor 2 (CXCR2 Gene) + 785C/T (rs2230054)

Chemokine receptors are cytokine receptors found predominantly on the surface of leukocytes that interact with a type of cytokine called a chemokine. Each has a rhodopsin-like seven-transmembrane structure and couples to G-protein for signal transduction within a cell, making them members of a large protein family of G protein-coupled receptors [15]. This gene is located in the chemokine receptor gene cluster region, including CCR1, CCRL2, CCR3, CCR5, and CCXCR1 on chromosome 3p [16]. As a proinflammatory cytokine, Interleukin-8 exerts immune and inflammatory actions through two chemokine receptors (CXCR1 and CXCR2) expressed by several leukocytes, including neutrophils and mast cells. IL-8 (CXCL8) is also termed neutrophil-activating protein-1 (NAP-1) [17]. CXCR2 (rs2230054) gene polymorphisms are linked to susceptibility to several diseases, including stroke induced by essential hypertension, prostate cancer, and systemic sclerosis [18]. 

Stokkeland et al. [19] reported increased serum CXCL8 and CXCL10 levels at week 10 of pregnancy in PCOS women, whereas Hatziagelaki et al. [20] reported that serum CXCL11 levels were strongly associated with prolactin and 17-OH-progesterone levels in PCOS. Other chemokines, like CXCL1~CXCL3, also contributed to the PCOS pathogenesis [21]. Recently, Ningning et al. [22] reported that the CXCL10, CXCR1, CXCR2, CXCL11, and CXCL8 expression levels were all significantly reduced in the fetal side placental tissue in the PCOS group. CXCR2 (rs2230054) and CXCR2 (rs1126580) are the two most common SNPs, and the SNP-derived gene variants could alter the gene transcription or the function of the encoded protein responsible for the pathogenesis of a disease. Studies on both humans and animals have linked CCR5 and CXCR2 to insulin resistance, adipose tissue inflammation, and obesity [23]. According to a different recent study, women with PCOS had considerably greater levels of CCR5 expression in circulating blood (WBC) cells and adipose tissue than women in the control group [24]. CCR5, CCL5, and CXCR2 expressions were significantly higher in the pgWAT mice (letrozole-treated) compared with the normal group of the mice [25]. 

### 1.3. Deletion of CCR5Δ32 in CC Chemokine Receptor 2 (CXCR2 Gene) 

The CC chemokine receptor 5 (CCR5) represents one of the chemokine receptor subtypes expressed by various immune cells involved in inflammatory responses. CCR5Δ32 polymorphism (rs333) is reported in the CXCR2 gene. CCR5 wild-type protein (CCR5-wt) consists of 352 amino acids and is folded into seven membrane-spanning domains connected by three extracellular and three intracellular loops. Most of the studies suggest that CCR5-delta32 polymorphism is linked to the risk of HIV-1 infection [26]. In addition to the wild type, a CCR5 allele with a 32 bp deletion (CCR5-Δ32) in the coding region is prevalent. Its product is a truncated protein that cannot be detected on the cell surface [27]. Moreover, there is a 32-base-pair (bp) deletion of the CCR5 gene coding region in the CCR5Δ32 variant. The CCR5Δ32 polymorphism (rs333) is a genetic variant that originated in the European population [28] and therefore can be used as an ancestry-informative marker in studies involving population genetics and genome ancestry [29]. This variant represents a 32-base-pair deletion in the *CCR5* gene (chromosome 3; 3p.21.31), a fundamental component of the immune system responsible for encoding the CCR5 protein, which acts mainly in the regulation of inflammatory cell migration. It is unclear what selective pressures (considering positive selection) were responsible for fixing CCR5Δ32 in the human genome. Smallpox, bubonic plague, and other infectious diseases have already been suggested, but there is no consensus on this aspect [30]. Neutral evolution is also a possibility [31]. What is relatively certain is that the variant probably originated in the European population 700–5000 years ago, potentially even earlier, and later spread heterogeneously across the world [32]. In animal influenza models, CCR5 plays a function in guiding CD8+ T lymphocytes to the site of infection, and its loss is related to higher death rates [33]. Findings indicate that among white individuals in the 2009 pandemic virus (H1N1), CCR5Δ32 was one of the variables linked to a higher degree of sickness [34].

## 2. Methodology 

### 2.1. Study Participants and Criteria

Protocols developed in accordance with the 2003 Rotterdam criteria were used to confirm PCOS cases. Non-Arabs, non-Saudis, and expatriates were not included in the study, but Saudi Arabs were. The study obtained 220 subjects from the Molecular Biology Laboratory (PFBSRC), Department of Medical Lab Technology, Faculty of Applied Medical Sciences, University of Tabuk. Patient specimens were collected from different hospitals in Tabuk City, King Fahd Special Hospital, and King Salman Military Hospital. We included 110 PCOS patients with clinical confirmation and 110 gender-matched controls.

### 2.2. Biochemical Serum Profile 

In its initial phase, the study looked at the biochemical serum profiles of the participants, including their insulin level, HbA1c, fasting glucose, serum lipids, and hormones. Using a hexokinase kit (Cobas Integra 800; Roche, Munich, Germany), the fasting glucose level was determined. ELISA kits were used for every evaluation. Estradiol, testosterone, TSH, FSH, LH, and progesterone were among the hormones whose blood levels were measured using ELISA kits that were standardized for each evaluation. Serum triglycerides, LDL, HDL, and total cholesterol were measured using a colorimetric determination (Integra 800; Roche). In accordance with the vendor’s instructions, an ELISA kit (DRG-EIA) was utilized to test the total insulin. Using a HOMA calculator, the HOMA-IR index was calculated (www.dtu.ox.ac.uk/homa/index, 25 January 2022). 

### 2.3. Extracting and Evaluating Genomic DNA Qualitatively

Following the vendor’s instructions, our lab extracted genomic DNA from peripheral blood samples of the patients and healthy controls using a DNA extraction kit (Cat # 69506/Qiagen, Hilden, Germany). Finally, the isolated DNA was dissolved in nuclease-free water and kept for later use at 4 °C. Using a NanoDropTM (Thermo Scientific, Waltham, MA, USA), the DNA was quantified. The extracted DNA was then qualitatively evaluated optically using the ratio of A260 nm/A280 nm (1.70–1.93).

### 2.4. Genotyping of TNF-α G>A (rs1800629), CXCR2-rs2230054-C>T CCR5-Delta32 rs333 Mutation

The genotyping of TNF-α-rs1800629 G>A, chemokine receptor 2-CXCR2-rs2230054-C>T, was determined by using amplification-refractory mutation system PCR and chemokine receptor 5 (CCR5) Delta32 mutation was determined by MS-PCR. Previously published ARMS primers were used TNF-α-rs1800629 G>A [35,36], chemokine receptor 2-CXCR2-rs2230054-C>T [32], and chemokine receptor 5 (CCR5) Delta32 mutation [37]. ARMS primers and MS primers are depicted in Table 1.

### 2.5. Preparation of PCR Mix

The PCR reaction was carried out in a reaction vol. of 12 μL, which was composed of four primers: Fo (0.10 μL), Ro (0.10 μL), FI (0.10 μL), RI (0.10 μL) (25 pmol of each primer), and 6 μL of green PCR Master Mix (2×) (Cat M712C) (Promega, Madison, WI, USA). The final volume of 12 μL was achieved by using nuclease-free ddH_2_O. Template DNA (50 ng) was added at the end. 

### 2.6. PCR Thermocycling Conditions

The thermocycling was as follows: initial denaturation at 95 °C for 11 min, followed by 30 cycles of denaturation at 94 °C for 35 s; annealing at 62 °C for 35 s (TNF-α 62 °C), annealing at 63 °C for 35 s (CXCR2+785C>T 63°C), annealing at 59.3 °C for 35 s (CCR5 Δ32 bp 59.3 °C); extension at 72 °C for 34 s, then final extension at 72 °C for 8 min and storage at 4 °C.

### 2.7. Gel Electrophoresis and PCR Product Visualization

PCR-amplified products were separated on 2% agarose gel electrophoresis, stained with sybre safe dye, and visualized under a UV transilluminator from Bio-Rad, Hercules, CA, USA.

**Figure 1:**
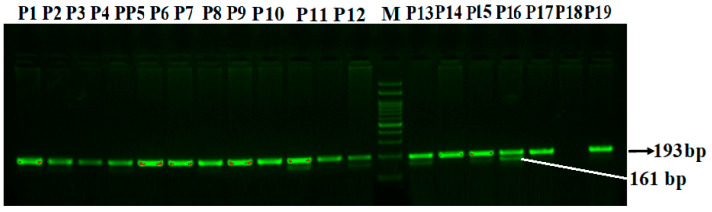
Agarose gel electrophoresis of PCR products corresponding to mutation-specific PCR for CCR5 Δ32 bp deletion in PCOS patients. CCR5 Δ32 bp deletion: M-100 bp DNA ladder; Homozygous CCR5 Δ32 (+/+): P1, P2, P3, P4, P5, P6, P7, P8, P9, P11, P14, P17, P19; Heterozygous CCR5 Δ32 +/Δ P10, P12, P13, P15, P16. CCR5 Δ32; Homozygous Δ/Δ – 0.

Mutation-specific PCR for CCR5 Δ32 bp:

The CCR5 gene is located on the short arm of chromosome 3 in the chemokine receptor gene cluster region. CCR5 Δ32 is a deletion of a gene that results in a non-functional receptor form of the chemokine receptor that is unable to bind CC chemokine ligands such as CCL5. PCR products were visualized by electrophoresis on a 3% agarose gel and classified on the basis of fragment length (168 bp for wild type and 193 bp for the CCR5 delta32 allele) (Figure 1).

**Figure 2:**
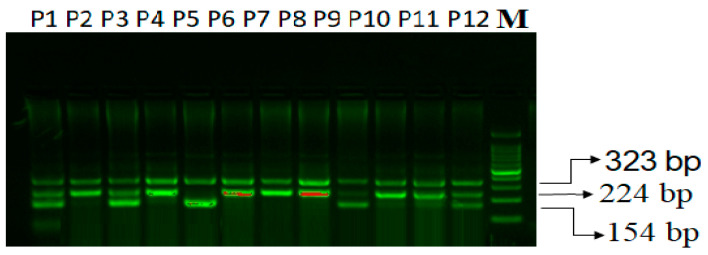
Agarose gel electrophoresis of PCR products corresponding to TNF-α rs1800629 G>A genotyping in PCOS patients. TNF-α rs1800629 G>A genotyping: M-100 bp DNA ladder; Homozygous-GG -P2, P4, P6, P7, P8, P9, P10, P11; Heterozygous-GA-P1, P3, P9, P12; Homozygous-AA-P5.

TNF-α rs1800629 G>A genotyping:

The TNF-α outer region is amplified by the outer primers Fo and Ro, yielding a band of 323 bp that serves as a DNA purity check. A band of 224 bp is produced by primers Fo and RI, amplifying the G allele, and a band of 154 bp is produced by primers FI and Ro, amplifying the A allele (Figure 2)**.**

**Figure 3:**
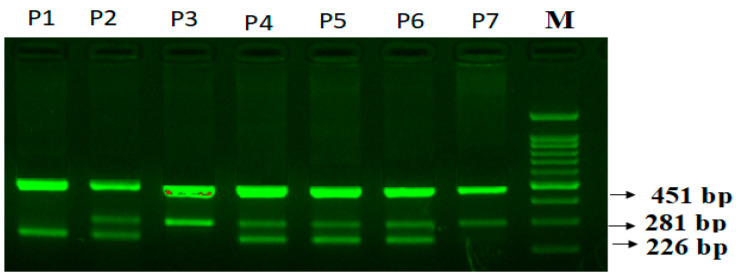
Agarose gel electrophoresis of PCR products corresponding to CXCR2+785C>T genotyping in PCOS patients. CXCR2+785C>T (rs2230054) genotyping: M-DNA ladder-100 bp; Homozygous-CC-P1; Heterozygous CA-P2, P4, P5, P6; Homozygous-AA-P3, P7.

CXCR2+785C>T (rs2230054) genotyping:

Primers Fo and Ro flanking the exon of the CXCR2+785C>T generated a band of 451 bp that served as a control for DNA integrity. Primers Fo and RI amplified the T allele, generating a band of 281 bp, and primers FI and Ro generated a band of 226 bp from the C allele, as shown in (Figure 3). 

### 2.8. Statistical Analysis:

The group distinctions were evaluated using Student’s two-sample *t*-test or one-way analysis of variance (ANOVA) for continuous variables and chi-squared (χ^2^) for categorical variables such as deviations from Hardy–Weinberg disequilibrium (HWD). Allelic and genotypic frequencies between the TNF-α G>A, CXCR2-C>T, and CCR5 Δ32 bp ins/del gene groups were evaluated using the chi-square test. The associations between tumor necrosis factor-α (TNF-α G>A (rs1800629)), chemokine receptor 5 (CCR5) Delta32 mutation, and chemokine receptor 2-CXCR2-rs2230054-C>T genotypes, and PCOS cases were evaluated by estimating risk ratios (RRs), odds ratios (ORs), and risk differences (RDs) through 95% confidence intervals. A *p*-value of <0.05 was considered significant. All statistical analyses were performed using Graph Pad Prism 8.4 and SPSS 16.

## 3. Results 

### 3.1. Demographic Characteristics of the Study Population

The study cohort was composed of 210 subjects, of which 110 were confirmed cases of PCOS, and 110 were matched healthy controls. Because PCOS is a complicated disorder, its effects are seen in a variety of clinical biomarkers that are considerably affected in PCOS patients. The demographic features of both patients and controls are presented in Table 2. As shown in Table 2, the majority of the measured biomarkers in patients revealed a significant change when compared with healthy controls. Patients had greater fasting glucose and insulin levels, indicating that they acquired T2DM and insulin resistance as the condition progressed. 

At the time of study inclusion, the mean age of the PCOS patient group was around 26.90 years, whereas the control group was 28.60 years. In PCOS patients, the majority of the biochemical markers that were examined in the research showed significant differences. In patients with notable deviations from the control groups, the lipid profile revealed elevated levels of serum cholesterol, TAGs, LDL, and HDL. In the patient group, there were notable changes in the levels of luteinizing hormone, progesterone, and follicle-stimulating hormone. In PCOS individuals who showed hyperandrogenism, a hallmark of this endocrine and metabolic condition, the level of testosterone was substantially higher. There were notable variations in the patients’ mean body mass index, which were linked to their changed lipid profiles.

#### Hardy–Weinberg Equilibrium (HWE) 

This research study has shown that the tumor necrosis factor-alpha (TNF-α) rs1800629 G>A frequency among the control subjects is in compliance with the HWE. The genotype distributions and allele frequencies of the SNPs located in the TNF-α rs1800629 G>A indicated no deviation in the Hardy–Weinberg equilibrium (HWE) in the polycystic ovary syndrome case group (all *p*-value > 0.05) (χ^2^ = 0.002, *p* ≤ 0.96) or in the matched healthy control group (*p* > 0.05) (χ^2^ = 0.20, *p* ≤ 0.64). In order to evaluate the genotyping results, 10% of the normal control group’s samples were selected at random, demonstrating a higher than 99% accuracy rate. Similar results were reported with CXCR2 rs2230054 C>T.

### 3.2. Allele and Genotype Frequency of TNF-α rs1800629 G>A and CXCR2 rs2230054 C>T Gene Polymorphism in Cases and Controls

The frequencies of tumor factor-alpha (TNF-α) rs1800629 G>A genotypes were as follows: in PCOS cases, GG (40.09%), GA (41.81%), and AA (9.09%); and in controls GG (70.90%), GA (27.27%), and AA (1.81%), respectively (Table 3). The TNF-α rs1800629 G>A gene variation observed between PCOS patients and controls was statistically significant (*p* < 0.001). Moreover, the frequency of the A allele was found to be higher among PCOS patients than in HC (0.59 vs. 0.41) (Table 3). The frequencies of CXCR2 rs2230054 C>T genotypes in PCOS patients and controls were as follows: in PCOS patients, CC (37.27%), GT (49.09%), and TT (13.63%); and in controls, GG (57.27%), CT (36.36%), and AA (6.36%), respectively (Table 3). It was noted that the distribution of CXCR2 rs2230054 C>T genotypes in PCOS patients and controls was significantly different (*p* = 0.008). However, the frequency of the T allele (fT) was significantly higher among patients (0.38 vs. 0.24) compared to controls, whereas the frequency of the C allele (fC) was lower in patients (0.62 vs. 0.76) compared to the controls, as exhibited in Table 3.

#### 3.2.1. Logistic Regression Analysis of TNF-α rs1800629 G>A Genotypes to Predict the Risk of PCOS Susceptibility 

An unconditional logistic regression was used to estimate associations between the genotypes and the risk of PCOS patients (Table 4). Logistic regression analysis was performed to determine the adjusted odds ratios (ORs) and 95% confidence intervals (95% CI) associated with the risk of PCOS after controlling for several covariates, taking the control women as the reference group. Our findings showed a strong link between TNF-α GA as well as TNF-α AA genotypes and PCOS predisposition in the codominant model (TNF-α-GG vs. AA) with an OR of 2.21, *p* < 0.006, and (TNF-α-GG vs. GA) with an OR of 7.22, *p* < 0.012 (Table 4). With an OR of 5.54, RR 1.62, and *p* < 0.032, the TNF-α (GA+AA) genotype vs. tumor necrosis factor-alpha (TNF-α)-GG genotype was correlated with PCOS risk in the dominant model. A strong correlation was reported between the TNF-α rs1800629 AA and TNF-α-(GA+GG) genotypes and PCOS with OR = 5.14 (95% CI: 1.08 to 24.33), RR = 3.11 (95% CI: 0.8036 to 11.114), and *p* < 0.038 in the recessive model. TNF-α-A allele was associated with PCOS susceptibility in the allelic model, with an OR of 2.34 (95% CI: 1.47 to 3.73), RR 1.60, and *p* < 0.0003 (Table 4).

#### 3.2.2. Logistic Regression Analysis of CXCR2+785 C>T (rs2230054 C>A) Genotypes to Predict the Risk of PCOS Susceptibility 

Our results indicated that in the codominant model, the heterozygosity CXCR2-CA genotype was correlated with an increased risk of PCOS, with an odds ratio of 1.91 (95% CI = 1.08–3.37), RR = 1.42 (1.0743 to 1.886), *p* < 0.024. With an OR of 3.29, RR 1.90, and *p* < 0.017, the CXCR2-AA genotype was strongly associated with PCOS susceptibility in the codominant inheritance model (Table 5). The results showed that in the dominant model, the CXCR2 (CA+AA) vs. CC genotype was associated with increased susceptibility to PCOS, with an OD 2.25 (95% CI = 1.131–3.872), RR = 1.49 (1.141 to 1.957), *p* < 0.032. A non-significant association was reported between the CXCR2-(CA+CC) vs. CXCR2 AA genotypes and PCOS susceptibility, with an OR = 2.32 (95% CI: 0.908 to 5.944), RR = 1.63 (95% CI: 0.874 to 3.058), and *p* < 0.078 in the recessive model. With an odds ratio of 1.89 (95% CI: 1.259 to 2.861), RR 1.40, and *p* < 0.002, the CXCR2-A allele was associated with PCOS susceptibility in allelic comparison (Table 4).

#### 3.2.3. CCR5Δ32 Allele Frequency (rs333) in PCOS Cases and Healthy Controls

The cystine–cystine chemokine receptor 5 (CCR5) is the primary HIV co-receptor involved in the viral entry process into human cells. The 32 bp deletion variant within the CCR5 gene (CCR5-Δ32) plays a very important role in viral recognition. The frequency of CCR5 Δ32 bp (rs333) genotypes in PCOS patients and controls was CCR5Δ32 (59.095%), CCR5(Δ32+Δ32*) (31.81%), and CCR5(Δ32*) (0%) for PCOS patients; and CCR5Δ32 (99%), CCR5(Δ32+Δ32*) (1%), and CCR5(Δ32*) (0%) for controls, respectively (Table 6). The CCR5 Δ32 gene mutation reported between PCOS cases and matched controls was significant (*p* < 0.0.0001). (Table 6).

#### 3.2.4. Logistic Regression Analysis of CCR5 Δ32 bp (rs333) Genotypes to Predict the Risk of PCOS Susceptibility 

Our findings showed a strong link between CCR5 (WT+Δ32*) genotypes with the PCOS predisposition in the codominant model, with an OR of 58.69 and RR of 22.25, *p* < 0.0001 (Table 7). With an OR of 58.69, an RR of 22.25, and *p* < 0.0001, the CCR5(WT+Δ32*) + CCR5(Δ32*) genotype vs. CCR5 (WT) genotype was linked with PCOS risk in the dominant model. With an odds ratio of 47.02 (95% CI: 6.37 to 346.81), RR 20.6, and *p* < 0.002, the CCR5Δ32* (mutant) allele was associated with PCOS susceptibility in allelic comparison (Table 7).

## 4. Discussion

In reproductive medicine, polycystic ovary syndrome (PCOS) is perhaps the most prevalent clinical diagnosis; however, it is still not well understood and is a major cause of female infertility, contributing to long-term complications associated with oxidative stress and chronic low-grade inflammation [2,3,38]. Improving the follicular milieu and reducing ovarian dysfunction might be achieved by managing chronic low-grade inflammation and fibrosis in the ovary. Kisspeptin is a protein that promotes the start of puberty, enhances the release of GnRH pulsatile during ovulation, and plays a critical role in the function of KNDy neurons, which are essential for the GnRH pulsatile signal necessary for reproduction.

### 4.1. Comparative Association of TNF-α rs1800629 G>A Gene Polymorphism 

Functional single nucleotide polymorphism (SNP) at positions −308 (rs1800629) of the TNF-α gene has been shown to be linked with altered promoter activity with different plasma levels of TNF-α in healthy individuals [39,40]. Among these SNPs, the A allele of rs1800629 and the C allele of rs1799964 polymorphisms have been shown to be associated with increasing TNF-α expression [41]. The A allele of rs1800629 polymorphism has been identified to be involved with insulin-dependent diabetes, the development of insulin resistance, and increasing adiposity [42]. Further, Rice et al. (1998) suggested that TNF-α appears to inhibit follicle-stimulating hormone (FSH) induced estradiol secretion in small follicles of the human ovary, leading to anovulation [43]. A potential physiological role of rs1800629 G >A polymorphism has been identified in several studies [44], and many recent reporter gene studies demonstrated a significant effect on its transcriptional activity [45]. Variation in the TNF-α gene was reported to be linked with PCOS susceptibility in the Korean population. The T allele of this polymorphism appeared to confer protection against PCOS in our study, while it was the C allele in the Korean population [46]. Studies on the TNF-α gene in PCOS patients from Australian, Caucasian, and Indian populations revealed a lack of association of rs1800629 polymorphism [47]. However, the rs1799964 T>C polymorphism suggested a strong influence on PCOS, similar to the findings of others in Asian populations [48]. 

### 4.2. Comparative Association of CXCR2 rs2230054 C>A Gene Polymorphism 

With regard to the C>T polymorphism in the CXCR2 gene, our findings showed a significant association between the heterozygous CT genotype in the CXCR2 gene (rs2230054) and the development of PCOS, with a *p*-value equal to 0.024. The CXCR2 gene encodes a protein that acts as a receptor for interleukin 8 (IL8), which is activated during inflammation. It helps in mediating neutrophil accumulation in the inflammation site. Indeed, most PCOS patients undergo chronic inflammation, and most studies that explored the rs2230054 SNP in both the heterozygous (CT) and homozygous (TT) status found significant results with inflammatory conditions. For example, Yao et al. (2019) found that both genotypes CT and TT are highly associated with systemic inflammatory response syndrome (SIRS) [49]. Our result in the heterozygous genotype agrees with Qi et al. (2021), who found a significant linkage of the heterozygous mode of inheritance (CT) with peri-implantitis in the Chinese Han population. [50]. In addition, the CT genotype was also found to be associated with chronic obstructive pulmonary disease (COPD) in the Tatar population from Russia [51]. Furthermore, our study also found an association of the homozygous genotype (TT) with the development of PCOS. These findings are similar to the results of Arikan et al. [15], who found that the homozygous genotype is associated with Behcet Disease (BH) which is blood vessel inflammation throughout the body. Variants at CXCR1 and CXCR2 have been associated with susceptibility to cutaneous and mucocutaneous leishmaniasis in Brazil [52]. When considering the studies that found associations of the CT and TT genotypes of the CXCR2 rs2230054, along with the present study, it seems that this SNP is more likely to be associated with the inflammation that accompanies PCOS disorder rather than the disease itself. 

On the other hand, Wang et al. [53] found this SNP to be associated with a decreased risk of goat arthritis in the Chinese Han population. This contradiction with our result might be attributed to the differences in the genetic composition among populations. Single nucleotide polymorphisms could help in the prediction of susceptibility of disease; however, they need strong evidence to be used in clinical diagnostics.

### 4.3. Comparative Association of CCR5 Δ32 bp (rs333) Gene Mutation 

The primary cell receptor for CCL3, CCL4, and CCL5 chemoreceptors is CCR5. An allelic variation of the CCR5 receptor gene, CCR5 Δ32, is distinguished by a 32 bp deletion (CCR5 Δ32 32), which results in an inactive protein, and as a result, CCR5 protein expression is absent in homozygous people. The pro-inflammatory response and osteoclastogenesis are two processes that the chemokine receptor CCR5 contributes to in the pathophysiology of illness. The CCR5 gene has a loss-of-function mutation called CCR5Δ32 (rs333), which may affect the host response and the development of periodontitis. The CCR5 gene has a loss-of-function mutation called CCR5Δ32 (rs333), which may affect the host response and the development of disease. The deletion in the CCR5 gene leads to a frameshift mutation that affects the open reading frame. Our data show an association between rs333 and PCOS. The CCR5 Δ32 gene mutation reported between cases (PCOS) and matched controls was significant (*p =* 0.0001). Increased heterozygosity frequency was documented in cases compared to matched controls: CCR5(Δ32+Δ32*) (31.81%) vs. CCR5(Δ32+Δ32*) (1%) (Table 6). In the literature, no studies have been performed to assess the relationship between this SNP and PCOS. However, many studies have found an association with inflammatory conditions. For example, a study in India found an association of rs333 with Japanese Encephalitis (Deval et al., 2019) [54]. Another study found an association of this SNP with decreased risk of chronic and aggressive periodontitis (Cavalla et al., 2018) [55]. A study by Dieter et al. (2022) [56] found that rs333 is associated with protection from COVID-19 infection. This indicates no specificity of the SNP with PCOS or any other disease. However, all these SNPs we studied could be further studied to investigate their association with inflammation.

**Table 8:** Distribution of CCR5 (Δ32) genotype and allele frequency in different ethnic populations.

Populations	*N*=	CCR5 Wild	CCR5 (Δ32+Δ32*)	CCR5Δ32*	Allele Frequency (%)	Ref.
Brazilian population	120	112 (93.33%)	8 (6.66%)	0	6.7	[57]
Australian Ashkenazi Jewish background	937	697 (74.38%)	219 (23.37%)	21	0.14	[58]
Australian non-Jewish population	442	372 (84.16%)	67 (15.15%)	3	0.08	[58]
Alia, Sicily	19	18 (94.73%)	1 (5.26%)	0	2.6	[59]
Lübeck, northern Germany	20	15 (75%)	5 (25%)	0	12.5	[59]
Göttingen, central Germany	346	287 (82.94%)	54 (15.6%)	5	9.2	[59]
Goslar, central Germany	19	12 (63.15)	7 (36.84%)	0	18.4	[59]
Inuit (Canada)	40	40 (100%)	0 (0)	0	0.0	[60]
Central Asian native populations	107	106 (99.06%)	1 (0.93%)	0	0.5	[60]
Russian	53	43 (81.13%)	9 (16.98%)	1	10.4	[60]
Asian West Siberian native populations	104	86 (82.69%)	13 (12.5%)	5	11.1	[60]
India	396	384 (96.96%)	12 (3.4%)	0 (0%)	3.0	[60]
Greece	375	362 (96.53%)	13 (3.46%)	0 (0%)	3.5	[61]
Saudi Arabia	110	109 (99.09%)	1 (1%)	0 (0%)	0.90	

In our study, we observed an absence of the homozygous Δ32 allele; the frequency of heterozygotes in the control group was 1%, which was comparable to previous studies on Central Asian native populations and Australians of Ashkenazi Jewish background. Our data indicated that the heterozygous carriers of the CCR5 (+/Δ) gene have 3.5-fold increased risk of recurrent miscarriages compared to the control group. Additionally, no person was discovered to be homozygous for the CCR gene mutation (allele), which is similar to many other groups under study (Table 8). Majumdar and Dey [62,63] found that the CCR5 32 allele was lacking in most of India’s ethnic communities, with the exception of a few in the country’s north and west, where it may have been brought in by a Caucasian gene flow. 

Suggestions: It is advised that further longitudinal studies be conducted in diverse ethnic populations and with larger sample sizes. It is suggested that investigations of gene–environment interactions with large cohorts should employ contemporary sequencing and statistical tools to find and describe novel SNPs important to PCOS prediction. The main limitation of the study is the small sample size.

## 5. Conclusions 

Our study concludes that TNF-α rs1800629G>A, CXCR2-rs2230054C>T, and CCR5-Delta32 rs333 are potential loci for developing PCOS in the Tabuk population. These findings might eventually be useful in identifying and classifying those who are at risk for PCOS. To validate these results, it is advised that further longitudinal studies be conducted in diverse ethnic populations and with larger sample sizes.

## Figures and Tables

**Table 1:** Primers for TNF-α, CXCR2 genotyping, and CCR5-Delta32 rs333 mutation.

**ARMS PCR primers for TNF-α rs1800629 G>A genotyping**				
TNF-αF0		5′-ACCCAAACACAGGCCTCAGGACTCAACA-3′	62 °C	323 bp
TNF-αR0		5′-TGGAGGCAATAGCTTTTGAGGGGCAGGA-3′		
TNF-α FI A	A allele	5′-AGTTGGGGACACGCAAGCATGAAGGAT**A**-3′		154 bp
TNF-α RIG	G allele	5′-TAGGACCCTGGAGGCTAGACCCCGTAC**C**-3′		224 bp
**ARMS PCR primers for CXCR2+785 C>T (rs2230054)** **genotyping**				
CXCR2-Fo		5′-CTGCCTGTCTTACTTTTCCGAAGGACCG-3′	63 °C	451 bp
CXCR2-Ro		5′-TCTTGAGGAGTCCATGGCGAAACTTCTG-3′		
CXCR2-FI	C allele	5′-TCTTTGCTGTCGTCCTCATCTTCCTGAT**C**-3′		226 bp
CXCR2-RI	T allele	5′-AGGACCAGGTTGTAGGGCAGCCAGAA**A**-3′		281 bp
**Chemokine receptor 5 Δ32 mutation-(CCR5 Δ32 bp ins/del) rs333**				
F-CCR5 Δ32	wild type	5′-TGT TTG CGT CTC TCC CAG-3′	59.3 °C	193 bp
R-CCR5 Δ32	Deletion	5′-CAC AGC CCT GTG CCT CTT-3′		161 bp

**Table 2:** Demographic and laboratory analysis of lipid profile and endocrine markers between PCOS and control.

Characteristic	Controls ^X^	Cases ^X^	*p* ^Y^
Age			
BMI (kg/m^2^) ^Z^	28.60 ± 3.59	26.90 ± 6.30	<0.0023
Age ^Z^	25.80 ± 5.16	27.55 ± 5.60	0.345
Triglycerides (mmol/L) ^Z^	1.80 ± 0.69	3.33 ± 1.75	0.046
LDL (mmol/L) ^Z^	2.98 ± 0.60	5.55 ± 1.69	<0.0014
Cholesterol (mmol/L) ^Z^	1.70 ± 0.55	1.78 ± 0.80	<0.0018
HDL (mmol/L) ^Z^	1.90 ± 0.80	1.92 ± 0.89	<0.0015
Progesterone (ng/mL) ^A^	15.80 (2.63–19.34)	19.50 (1.75–35.80)	<0.0031
Luteinizing hormone (mIU/mL) ^A^	0.08 (0.07–1.60)	3.60 (0.68–8.62)	<0.0024
Testosterone (ng/dL) ^A^	15.44 (7.90–14.25)	62.56 (45.40–90.49)	<0.0031
Estradiol (pmol/L) ^A^	25.88 (141.32–520.10)	236.40 (180.21–544.21)	0.267
FSH (mIU/mL) ^A^	0.80 (0.61–4.40)	5.80 (2.65–7.08)	<0.0032
Fasting blood sugar FBS (mmol/L) ^Z^	4.78 ± 0.79	8.70 ± 4.61	<0.0030
HOMA-IR ^Z^	3.05 ± 0.80	5.12 ± 4.70	<0.0024
Free Insulin (mU/mL) ^Z^	7.80 ± 2.90	14.19 ± 5.90	<0.0031

^X^ 110 PCOS cases and 110 healthy controls; ^Y^ Student’s *t*-test (continuous variables); ^Z^ values as mean ± SD; ^A^ values presented as median (interquartile range); Mann–Whitney U-test (normally distributed variables).

**Table 3:** TNF-α rs1800629 G>A and CXCR2 rs2230054 C>A genotype distribution between cases and controls.

**Correlation Tumor Necrosis Factor-Alpha (TNF-α) rs1800629 G>A genotypes between cases and healthy controls**									
	*N*=	GG	GA	AA	G	A	Df	X^2^	*p*-value
PCOS	110	54 (40.09%)	46 (41.81%)	10 (9.09%)	0.41	0.59	2	13.07	0.001
Controls	110	78 (70.90%)	30 (27.27%)	02 (1.81%)	0.85	0.15			
**Association of CXCR2 rs2230054 C>T genotypes between cases and healthy controls**									
	*N*=	CC	TC	TT	C	T	Df	X^2^	*p*-value
PCOS	110	41 (37.27%)	54 (49.09%)	15 (13.63%)	0.62	0.38	2	9.65	0.008
Controls	110	63 (57.27%)	40 (36.36%)	07 (6.36%)	0.76	0.24			

**Table 4:** Multivariate analysis to estimate the association of TNF-α rs1800629 G>A genotypes to predict the risk of PCOS susceptibility.

Genotypes	Healthy Controls	PCOS Cases	Odd Ratio OR (95% CI)	Risk Ratio RR (95% CI)	*p*-Value
	(*N* = 110)	(*N* = 110)			
Codominant inheritance model					
TNF-α-(GG)	78	54	(ref.)	(ref.)	
TNF-α-(GA)	30	46	2.21 (1.2448 to 3.940)	1.49 (1.0952 to 2.046)	0.006
TNF-α-(AA)	02	10	7.22 (1.5217 to 34.278)	3.54 (0.9926 to 12.664)	0.012
Dominant inheritance model					
TNF-α-(GG)	78	54	(ref.)	(ref.)	
TNF-α (GA+AA)	32	56	5.54 (1.1549 to 25.248)	1.62 (1.1910 to 2.217)	0.032
Recessive inheritance model					
TNF-α-(GA+GG)	108	100			
TNF-α-AA	02	10	5.14 (1.0867 to 24.337)	3.11 (0.8732 to 11.114)	0.038
Additive inheritance model (alleles)					
TNF-α-G	186	154	1 (ref.)	1 (ref.)	
TNF-α-A	34	66	2.34 (1.4718 to 3.734)	1.60 (1.2043 to 2.149)	0.0003
Overdominant inheritance model					
TNF-α-(GG+AA)	80	64	(ref.)	(ref.)	
TNF-α (GA)	30	46	1.91 (1.0890 to 3.373)	1.40 (1.0277 to 1.927)	0.024

**Table 5:** Multivariate analysis to estimate the association of CXCR2+785 C>T (rs2230054 C>A) genotypes to estimate the risk of PCOS.

Genotypes	Healthy Controls	PCOS Cases	OR (95% CI)	RR (95% CI)	*p*-Value
	(*N* = 110)	(*N* = 110)			
Codominant inheritance model					
CXCR2-CC	63	41	(ref.)	(ref.)	
CXCR2-CA	40	54	1.91 (1.0890 to 3.373)	1.42 (1.0743 to 1.886)	0.024
CXCR2-AA	07	15	3.29 (1.2363 to 8.769)	1.90 (1.0129 to 3.578)	0.017
Dominant inheritance model					
CXCR2-CC	63	41	(ref.)	(ref.)	
CXCR2 (CA+AA)	47	69	2.25 (1.3140 to 3.872)	1.49 (1.1418 to 1.957)	0.032
Recessive model					
CXCR2-(CA+CC)	103	95			
CXCR2-AA	07	15	2.32 (0.9080 to 5.944)	1.63 (0.8741 to 3.058)	0.078
Allele					
CXCR2-C	166	136	1(ref.)	1(ref.)	
CXCR2-A	54	84	1.89 (1.2598 to 2.861)	1.40 (1.1141 to 1.771)	0.002
Overdominant inheritance model					
CXCR2	70	56	(ref.)	(ref.)	
CXCR2-(CA)	40	54	1.68 (0.9842 to 2.893)	1.30 (0.9847 to 1.731)	0.057

**Table 6:** Association of CCR5 Δ32 bp (rs333) genotypes with PCOS susceptibility.

Subjects	*N*=	Wild Genotype CCR5 Wild	Heterozygous Mutant CCR5 (Δ32+Δ32*)	Mutant Genotype CCR5Δ32*	Df	CCR5 Δ32	CCR5Δ32*	X^2^	*p*-Value
Cases	100	65 (59.09%)	35 (31.81%)	0	2	0.84	0.16	46.86	0.0001
Controls	110	109 (99%)	1 (1%)	0		0.99	0.1		

**Table 7:** Multivariate analysis to estimate the association of CCR5 Δ32 bp (rs333) genotypes with PCOS susceptibility.

Genotype	Controls	PCOS	OR (95% CI)	OR (95% CI)	*p*-Value
Codominant inheritance model					
CCR5(WT)	109	65	**(ref.)**	**(ref.)**	
CCR5(WT+Δ32*)	01	35	58.69 (7.85 to 438.65)	22.25 (3.253 to 156.303)	0.0001
CCR5Δ32*(mutant)	0	0	0	0	
Dominant inheritance model					
CCR5(WT)	109	65	**(ref.)**	**(ref.)**	
CCR5(WT+Δ32*) + CCR5(Δ32*)	01	35	58.69 (7.85 to 438.65)	22.25 (3.253 to 156.303)	0.0001
Recessive inheritance model					
CCR5(WT) + CCR5(WT+Δ32*)	110	98	**(ref.)**	**(ref.)**	
CCR5Δ32*(mutant)	0	0	0	0	0
Allele			**(ref.)**	**(ref.)**	
CCR5(WT)	219	163			
CCR5Δ32*(mutant)	1	35	47.02 (6.37 to 346.81)	20.6 (2.982 to 142.83)	0.002

## Data Availability

All data associated with the research study are presented in the manuscript.
